# A Unique High‐Output Cardiac Hypertrophy Phenotype Arising From Low Systemic Vascular Resistance in Cantu Syndrome

**DOI:** 10.1161/JAHA.122.027363

**Published:** 2022-12-14

**Authors:** Gautam K. Singh, Conor McClenaghan, Manish Aggarwal, Hongjie Gu, Maria S. Remedi, Dorothy K. Grange, Colin G. Nichols

**Affiliations:** ^1^ Division of Cardiology, Department of Pediatrics Washington University School of Medicine St. Louis MO; ^2^ Center for the Investigation of Membrane Excitability Diseases (CIMED) Washington University School of Medicine St. Louis MO; ^3^ Department of Cell Biology and Physiology Washington University School of Medicine St. Louis MO; ^4^ Division of Statistics Washington University School of Medicine St. Louis MO; ^5^ Department of Medicine, Division of Endocrinology Washington University School of Medicine St. Louis MO; ^6^ Department of Pediatrics, Division of Genetics Washington University School of Medicine St. Louis MO

**Keywords:** *ABCC9*, cardiomegaly, echocardiography, heart failure, high‐output state, Ion Channels/Membrane Transport, Cardiomyopathy, Congenital Heart Disease, Heart Failure, Hypertrophy

## Abstract

**Background:**

Cardiomegaly caused by left ventricular hypertrophy is a risk factor for development of congestive heart failure, classically associated with decreased systolic and/or diastolic ventricular function. Less attention has been given to the phenotype of left ventricular hypertrophy with enhanced ventricular function and increased cardiac output, which is potentially associated with high‐output heart failure. Lack of recognition may pose diagnostic ambiguity and management complexities.

**Methods and Results:**

We sought to systematically characterize high‐output cardiac hypertrophy in subjects with Cantu syndrome (CS), caused by gain‐of‐function variants in *ABCC9*, which encodes cardiovascular K_ATP_ (ATP‐sensitive potassium) channel subunits. We studied the cardiovascular phenotype longitudinally in 31 subjects with CS with confirmed *ABCC9* variants (median [interquartile range] age 8 years [3–32 years], body mass index 19.9 [16.5–22.9], 16 male subjects). Subjects with CS presented with significant left ventricular hypertrophy (left ventricular mass index 86.7 [57.7–103.0] g/m^2^ in CS, n=30; 26.6 [24.1–32.8] g/m^2^ in controls, n=17; *P*<0.0001) and low blood pressure (systolic 94.5 [90–103] mm Hg in CS, n=17; 109 [98–115] mm Hg in controls, n=17; *P*=0.0301; diastolic 60 [56–66] mm Hg in CS, n=17; 69 [65–72] mm Hg in control, n=17; *P*=0.0063). Most (21/31) subjects with CS exhibited eccentric hypertrophy with normal left ventricular wall thickness. Congestive heart failure symptoms were evident in 4 of the 5 subjects with CS aged >40 years on long‐term follow‐up.

**Conclusions:**

The data define the natural history of high‐output cardiac hypertrophy resulting from decreased systemic vascular resistance in subjects with CS, a defining population for long‐term consequences of high‐output hypertrophy caused by low systemic vascular resistance, and the potential for progression to high‐output heart failure.

**Nonstandard Abbreviations and Acronyms**
CO: cardiac output
CS: Cantu syndrome
HR: heart rate
ICSR: International Cantu Syndrome Registry
K_ATP_
: ATP‐sensitive potassium
LVMI: left ventricular mass index
PAAT: pulmonary artery acceleration time
RWT: relative wall thickness

**Clinical Perspective**

**What Is New?**

- Cantu syndrome (CS) is characterized by a high‐output state that may progress to heart failure with normal diastolic function, low systemic vascular resistance, and high output.
- Although CS and heart failure with preserved ejection fraction share the general symptoms of heart failure, they are distinct in cardiac phenotype and pathophysiology, and CS should be considered in the differential diagnosis of heart failure where ventricular systolic function appears normal, such as heart failure with preserved ejection fraction.

**What Are the Clinical Implications?**

- Because of a current lack of awareness of CS complications, many subjects with CS have been misdiagnosed with hypertrophic cardiomyopathy or dilated cardiomyopathy, and as a result have been administered conventional therapies including angiotensin‐converting enzyme inhibitors, β‐blockers, or sildenafil, but treatment with these agents is based on heart failure paradigms that do not take into account low systemic vascular resistance states and may exacerbate the underlying primary decrease in systemic vascular resistance and hence worsen symptoms.

Left ventricular hypertrophy (LVH) without structural disorder of the heart represents a risk factor for development of heart failure (HF).^1^ Initially, LVH may serve to maintain contractile forces and counteract increased ventricular wall stress caused by altered loading conditions. However, increased wall stress may lead to apoptosis, myocardial fibrosis, and architectural changes that are associated with decline in systolic reserve capacity and diastolic function.^2^ Thus, LVH serves as a risk factor and structural prerequisite for the development of HF from impaired pump function that can progress to left ventricular (LV) remodeling without symptoms of HF and then to symptomatic congestive HF, a low‐output state. This current clinical HF paradigm of an LVH phenotype with impaired pump function and low cardiac output (CO) is predominantly cardiomyocentric. In current clinical practice, this essentially excludes primary vasculopathies that may be involved in the HF pathogenesis, including any LVH that may be related to a primary vascular pathology with the potential to progress to congestive HF. Relatively little is understood about the underlying mechanisms and natural history of high‐output cardiac hypertrophy states arising from primary vascular pathology. Such lack of awareness could result in ambiguity of clinical diagnosis, adversely influencing clinical screening and management.

We describe the natural history of a cardiac phenotype characterized by LVH with enhanced ventricular function and high‐output state caused by genetically determined vasodilatation, in a cohort of subjects with Cantu syndrome (CS; Online Mendelian Inheritance in Man number 239850), an autosomal dominant condition caused by gain‐of‐function variants in *ABCC9* and, less commonly, in *KCNJ8*, which encode the regulatory (Sulfonylurea receptor 2 [SUR2]) and pore‐forming (inwardly rectifying potassium channel family member 6.1) subunits, respectively, of K_ATP_ (ATP‐sensitive potassium) channels.^3^, ^4^, ^5^, ^6^ Subjects with CS exhibit a wide constellation of clinical features,^7^, ^8^, ^9^ with congenital hypertrichosis, osteochondrodysplasia, and characteristic facies. We describe the longitudinal cardiovascular phenotype in 31 subjects with CS and discuss the potential mechanistic basis, natural history of the high‐output state, and the clinical implications of this sentinel example of high‐output cardiac hypertrophy associated with primary decreased systemic vascular resistance.

## METHODS

### Study Design and Data Collection

We studied the cardiovascular phenotype in 31 subjects with CS with confirmed *ABCC9* variants.^9^ All subjects were enrolled in the ICSR (International Cantu Syndrome Registry), established as an international multicenter Research electronic data capture‐based registry to collate systematic clinical and genetic data on subjects with CS. Longitudinal data were collected during annual CS research clinics over 7 years from 2013 to 2019.^9^ Initial cardiovascular findings in the first 11 subjects with CS studied in the Washington University clinic have been reported previously.^10^, ^11^ Cardiovascular phenotype in 17 age‐ and sex‐matched normal healthy subjects were included as controls. Medical history and prior medical records, including results of molecular genetic testing, were collated in questionnaire form for each subject, either during routine visits as part of standard care or during the annual CS research clinic. All studies were approved by the Washington University School of Medicine Institutional Review Board of the Human Research Protection Office and performed at St. Louis Children's Hospital. All data were collected after informed consent of the subjects or of the parents or legal guardians for subjects aged <18 years. The data that support the findings of this study are available from the corresponding authors upon reasonable request.

### Cardiovascular Examination

Cardiovascular examination included detailed history, tracking of symptoms, complete physical examination, and standard vital measurements.

#### Blood Pressure

Blood pressure (BP) and heart rate (HR) were measured 3 times by manual sphygmomanometer with appropriate cuff size, in the resting supine position, and after 2 and 5 minutes in the upright position, consistent with American Academy of Pediatrics guidelines,^12^ to ascertain orthostatic vital sign changes. BP measurements were compared with published normative values by sex and age (www.nhlbi.nih.gov/files/docs/guidelines/child_tbl.pdf).

#### Cardiac Structure

Transthoracic complete echocardiographic examinations were performed according to American Society of Echocardiography guidelines (Vivid 9 and E95; General Electric Medical Systems). M‐mode measured LV end‐diastolic diameter, LV end‐systolic diameter, and end‐diastolic posterior wall thickness were used to calculate relative wall thickness (relative wall thickness (RWT)=2×posterior wall thickness/LV end‐diastolic diameter)^13^ and LV mass. LV mass index (LVMI) was calculated and indexed to height using the Devereux equation.^14^ To define ventricular structural phenotype, the 95th percentiles for LVMI for children older than 9 years (>40 g/m^2.7^ in female children and >45 g/m^2.7^ in male children) and the 95th percentile value for RWT for normal children and adolescents (RWT>0.41) were used as cutoff values to categorize abnormal LV geometry.^15^, ^16^ Subjects with LVMI and RWT below the 95th percentile were categorized as having normal LV geometry. Concentric remodeling was defined as normal LVMI and elevated RWT, eccentric hypertrophy as elevated LVMI and normal RWT, and concentric hypertrophy as elevated LVMI and RWT.^17^ Right ventricular (RV) dimensions in apical 4‐chamber images were measured for basal and midcavity minor axis and longitudinal axis in RV focus view.

#### Cardiac Function

LV systolic function was measured in standard format using M‐mode imaging for LV fractional shortening and the 2‐dimensional imaging modified Simpson method for LV ejection fraction. Myocardial mechanics were evaluated by measurement of strain (dimensionless measure of myocardial deformation) and strain rate (time derivative of strain), which correlates with LV peak elastance, reflecting contractile performance.^18^ LV peak global longitudinal strain, strain rates, early diastolic global longitudinal strain rates, and late diastolic global longitudinal strain rates were measured using 2‐dimensional speckle‐tracking echocardiography (EchoPAC, General Electric Medical Systems). LV and RV systolic and diastolic functions were also evaluated using tissue Doppler imaging.^19^ Tissue Doppler imaging–measured indices were compared with the normal controls and with published normative values by sex and age.^20^

#### Cardiovascular Hemodynamics

CO was calculated from measured aortic valve annular diameter, time velocity integral of pulse‐wave Doppler velocity in LV outflow tract, and HR (beats per minute) using the equation CO=π×(diameter/2)^2^×time velocity integral×HR×100 (liters per minute), and indexed (cardiac index) against body surface area (liters per minute per square meter). Systemic vascular resistance (SVR) was calculated by the formula: SVR=mean arterial pressure/CO (mean arterial pressure [systolic BP+{2×diastolic BP}]/3) and CO (millimeters of mercury per minute per milliliter). Additionally, cardiac hemodynamics were studied in 5 subjects with CS by cardiac catheterization, in 2 subjects when they had either device closure (CS001) or before surgical ligation (CS005) of a patent ductus arteriosus (PDA), and in another 3 subjects who underwent cardiac catheterization for hemodynamic study (CS002 and CS004) or interventional embolization of a major aorto‐pulmonary collateral (CS020) as a part of standard of care.

#### Pulmonary Hemodynamics

Pulmonary artery acceleration time (PAAT) demonstrates a strong, inverse correlation with invasively measured pulmonary hemodynamics.^21^, ^22^ We obtained PAAT using pulse Doppler velocity, as previously reported, and standardized against HR with the ratio of PAAT/RV ejection time.^23^

#### Vascular Properties

M‐mode ultrasonography of the ascending aorta was used to assess arterial stiffness using the modified equation for calculation of Young's elastic modulus: Young's elastic modulus=(aortic radius in systole/arterial wall thickness)×(pulse pressure×fractional change in diameter of the aorta from end‐diastole to end‐systole).^24^

### Statistical Analysis

Data were analyzed by SAS version 9.4 (SAS Institute, Cary, NC). Quantitative data are presented as median (interquartile range), and qualitative data are presented as number (percent). In case–control comparisons, only values from the first clinic visit of each individual are included. A Wilcoxon rank sum test was used to assess differences between nonnormal or continuous variables of unknown distributions between groups. χ^2^ test or Fisher exact test (if the number for any group was <5) was used to test associations between categorical factors and groups. In longitudinal analysis, only subjects who had at least 2 visits were included. A mixed random‐effects repeated measures model was performed to describe the rate of change of continuous variables versus time. *P*<0.05 was considered as significant.

## RESULTS

### Demographic and Anthropometric Characteristics of the CS Cohort

Cardiovascular phenotype was initially assessed in 31 subjects with CS aged 1 to 69 years. The majority (20/31; 65%) were aged <18 years at the initial enrollment or evaluation for standard of care, and 16 (52%) were male subjects. The control group was similar in age, sex, race, and ethnicity distribution (Table 1). Anthropometric data were not significantly different between the 2 groups, except height, where the subjects with CS were shorter (Table 1).

**Table 1:** Demographic and Clinical Characteristics for Control and Case Individuals

	Control	Case	*P* value
Demographics			
Men, n (%) [N]	10 (58.82) [17]	16 (51.6) [31]	0.6316
Median age at the first visit, y, (IQR) [N]	15 (14–17) [17]	8 (3–32) [31]	0.4101
Race, n (%)			
White	15 (88.24)	30 (96.8)	0.1258
Black	2 (11.76)	0 (0)	
Not recorded	0 (0)	1 (3.2)	
Ethnicity, n (%)			
Hispanic or Latino	0 (0)	8 (25.8)	0.1638
Non‐Hispanic or Latino	8 (47.1)	22 (71.0)	
Not recorded	9 (52.9)	1 (3.2)	
Anthropometrics			
Median BMI (IQR) [N]	19.6 (18.1–20.4) [17]	19.9 (16.5–22.9) [30]	0.6363
Median weight, kg (IQR) [N]	53.3 (48.4–61.4) [17]	35.2 (18.9–60) [30]	0.2055
Median height, cm (IQR) [N]	166.8 (158.8–172.2) [17]	137.5 (102.3–162) [30]	0.0169
Median BSA (Du Bois) (IQR) [N]	1.62 (1.5–1.7) [17]	1.1 (0.7–1.7) [30]	0.1013

Quantitative data are presented as median (IQR), and qualitative data are presented as n (%). *P* values are from Wilcoxon rank sum test for continuous variables, or χ^2^ test or Fisher exact test (if any expected cell number <5) for categorical variables. *P*<0.05 indicates there is a significant difference between case and control. BMI indicates body mass index; BSA, body surface area; and IQR, interquartile range.

### Cardiovascular Phenotype in CS Subjects

**Figure 1:**
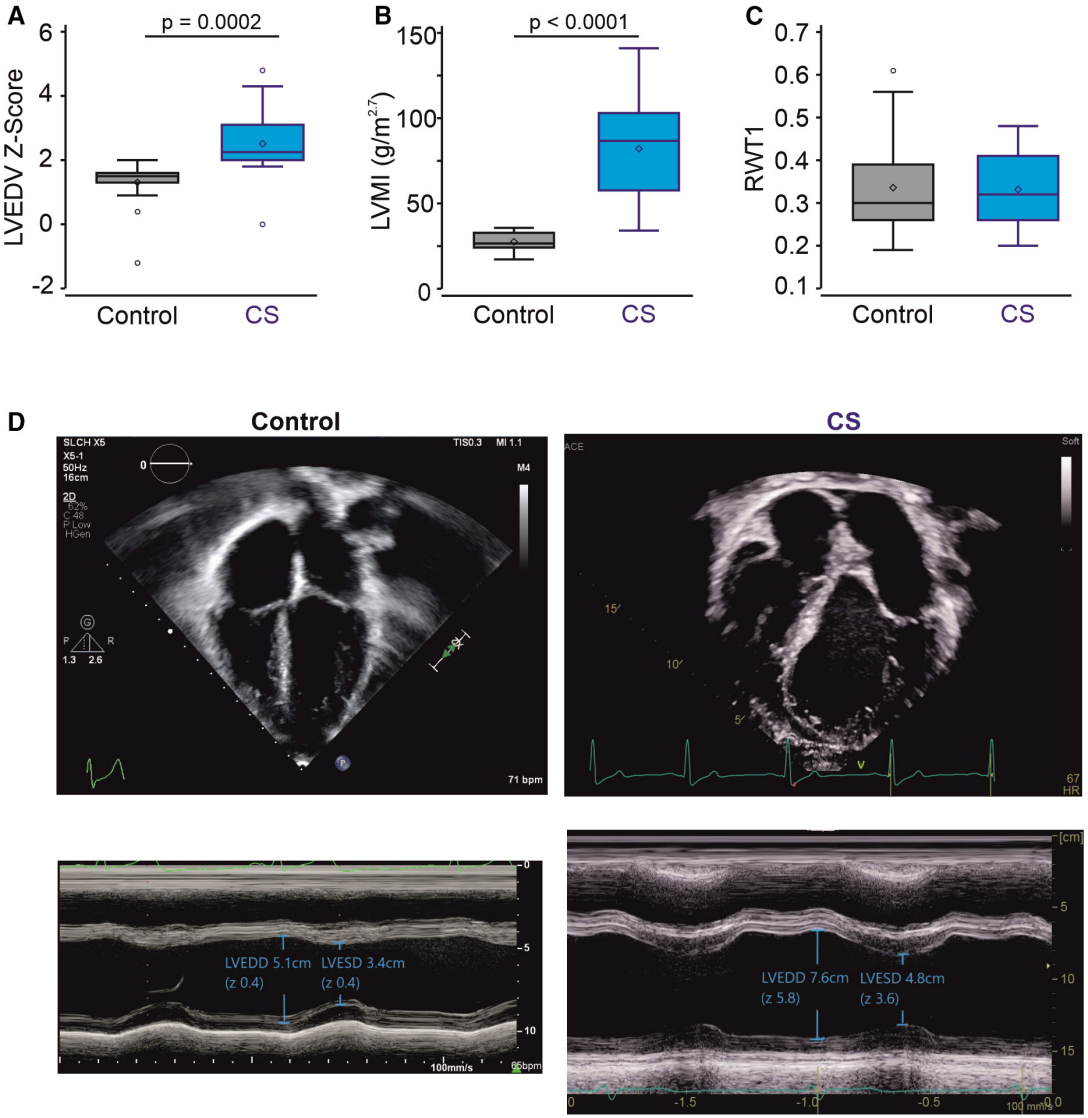
Structural remodeling in the CS heart. LVEDV *Z* score (**A**) and LVMI (**B**) are increased in subjects with CS compared with the control group. **C**, RWT1 is normal in subjects with CS. *P* values from Wilcoxon rank sum tests are shown. *P*<0.05 indicates a statistically significant difference between control and CS cohorts. **D**, Representative 4C (above) and M‐mode (below) echocardiographic images of a control (male sex, 64 kg, ages 15 years [left]) and CS (male sex, 77 kg, aged 16 years [right]) subjects. 4C end‐diastolic volumes were 116 mL (*Z*=−0.5) and 389 mL (*Z=*8.1), and ejection fractions were 59% and 70%, for the control and subject with CS, respectively. M‐mode dimensions were diastolic 5.1 cm (*Z=*0.4) and 7.6 cm (*Z=*5.8), systolic 3.4 cm (*Z=*0.4) and 4.8 cm (*Z=*3.6), and shortening fraction was 33% and 37%, respectively. CS indicates Cantu syndrome; LVEDD, left ventricular end diastolic dimension; LVEDV, left ventricular end‐diastolic volume; LVESD, left ventricular end systolic dimension; LVMI, left ventricular mass index; and RWTI, relative wall thickness.

All subjects with CS had normal cardiac segmental anatomy, and they had normal wall thickness on average, but all exhibited markedly enlarged hearts and LV volumes and mass (LVMI) throughout the course of the investigation, compared with normal controls (Figure 1A through 1D). The majority (21/31; 68%) of subjects were determined to exhibit eccentric hypertrophy with normal LV posterior wall thickness, despite increased LVMI (Table 2). A further 6 subjects (6/31; 19%) who exhibited concentric hypertrophy with increased LVMI and increased wall thickness (Table 2) were predominantly younger (all aged <18 years at presentation).

**Table 2:** Cardiac Function and Hemodynamics

	Control	Case	*P* value
LV structure and function			
Median LVEDV, mL, (IQR) [N]	91 (78 to 98) [17]	84.3 (53.3 to 165) [31]	0.066
Median *Z* score of LVEDV (IQR) [N]	1.5 (1.3 to 1.6) [17]	2.3 (2.0 to 3.1) [31]	<0.0001
Median LVDd, cm, (IQR) [N]	4.3 (4.0 to 4.5) [17]	4.4 (3.9 to 5.6) [31]	0.2172
Median *Z* score of LVDd (IQR) [N]	1.20 (0.90 to 1.80) [17]	2.4 (1.9 to 3.3) [31]	<0.0001
Median LVMI‐M‐mODE, g/m^2.7^, (IQR) [N]	26.6 (24.1 to 32.8) [17]	86.7 (57.7 to 103.0) [30]	<0.0001
LVMI abnormal, n (%)			<0.0001
Normal	17 (100)	3 (9.7)	
Abnormal	0 (0)	26 (83.8)	
Not recorded	0 (0)	2 (6.5)	
Remodeling, n (%)			
Normal	14 (82.3)	2 (6.5)	<0.0001
Concentric remodeling	3 (17.7)	6 (19.3)	
Eccentric hypertrophy	0 (0)	21 (67.7)	
Not recorded	0 (0)	2 (6.5)	
Median RWT1 (IQR) [N]	0.3 (0.26 to 0.39) [17]	0.32 (0.26 to 0.41) [31]	0.9035
RWT1 abnormal, n (%)	3 (17.7)	7 (21.2)	0.7271
Median FS, %, (IQR) [N]	36 (36 to 38) [17]	43.4 (40.4 to 46.6) [31]	<0.0001
FS abnormal, n (%)	0 (0)	11 (33.3)	0.0038
Median LV GS long, % overall, (IQR) [N]	−18.0 (−20.1 to −17.1) [17]	−24.3 (−25.4 to −23.1) [30]	<0.0001
LV GS long, % overall abnormal, n (%)	8 (47.1)	29 (87.9)	<0.0001
Median LV GSRs long, %/s (IQR) [N]	−1.0 (−1.1 to −0.9) [17]	−1.4 (−1.7 to −1.3) [22]	0.0001
Median LV GSRe long, %/s (IQR) [N]	1.6 (1.4 to 1.9) [17]	2.0 (1.7 to 2.5) [22]	0.0035
Median LV GSRa long, %/s (IQR) [N]	0.55 (0.48 to 0.82) [17]	0.87 (0.70 to 1.7) [22]	0.0025
Median LV E'/A', (IQR) [N]	2.9 (2.6 to 3.2) [17]	2.0 (1.7 to 2.5) [28]	0.0050
Median LV E/E', (IQR) [N]	6.3 (5.3 to 8.0) [17]	7.9 (6.0 to 9.0) [30]	0.1273
LV E/E' abnormal, n (%)			
Normal	10 (58.8)	15 (48.4)	0.2197
Borderline	4 (23.5)	14 (45.2)	
Abnormal	0 (0)	0 (0)	
Not recorded	3 (17.7)	2 (6.5)	
Median LV MPI (IQR) [N]	0.35 (0.32 to 0.38) [17]	0.37 (0.32 to 0.40) [30]	0.9897
LV MPI abnormal, n (%)	11 (64.7)	25 (75.8)	0.6646
Hemodynamics			
Median EF, %, (IQR) [N]	63 (59 to 65) [17]	68.5 (61 to 76) [31]	0.0124
EF abnormal, n (%)	4 (25.5)	20 (60.6)	0.0045
Median CI, L/min per m^2^, (IQR) [N]	4.20 (4 to 4.8) [17]	6.7 (5.5 to 7.6) [31]	0.0004
CI abnormal, n (%)	0 (0)	25 (75.8)	<0.0001
Median Young's elastic modulus, (IQR) [N]	14.8 (10.8 to 18.5) [17]	26.1 (16.6 to 43.4) [25]	0.0043
Median systolic pressure, mm Hg, (IQR) [N]	109 (98 to 115) [17]	94.5 (90 to 103) [17]	0.0301
Median diastolic pressure, mm Hg, (IQR) [N]	69 (65 to 72) [17]	60 (56 to 66) [17]	0.0063
Median systemic vascular resistance, (IQR) [N]	20 (18.8 to 21.1) [17]	9.5 (9.1 to 11.2) [17]	<0.0001
Median systolic AAO dimension, cm, (IQR) [N]	2.7 (2.5 to 2.8) [17]	2.3 (1.8 to 3.2) [29]	0.4841
Median *Z* score of systolic AAO dimension, (IQR) [N]	1.6 (1.3 to 1.8) [17]	2.9 (2.2 to 3.8) [29]	<0.0001
Median diastolic AAO dimension, cm, (IQR) [N]	2.5 (2.4 to 2.7) [17]	2.0 (1.5 to 2.8) [17]	0.2752
Median heart rate, bpm, (IQR) [N]	77 (72 to 89) [17]	93 (78 to 110) [31]	0.0317

Quantitative data are presented as median (IQR), and qualitative data are presented as n (%). *P* values are from Wilcoxon rank sum test for continuous variables, or χ^2^ test or Fisher exact test (if any expected cell number <5) for categorical variables. For other variables, *P* values were associated with mixed general linear model for longitudinal data analysis. *P*<0.05 indicates there is a significant difference between case and control. AAO indicates ascending aorta; CI, cardiac index; E'/A', early diastolic velocity of mitral annulus (E') over late diastolic velocity of mitral annulus (A'); E/E', early mitral peak inflow velocity (E) over early diastolic velocity of mitral annulus (E'); EF, ejection fraction; FS, fractional shortening; IQR, interquartile range; LV, left ventricular; LV GS long, left ventricular global longitudinal strain; LV GSRa, left ventricular global peak late diastolic strain rate; LV GSRe, left ventricular global peak early diastolic strain rate; LV GSRs, left ventricular systolic global strain rate; LV MPI, left ventricular myocardial performance index; LVDd, left ventricular diastolic dimension; LVEDV, left ventricular end‐diastolic volume; LVMI, left ventricular mass index; LVMI‐M‐mode, left ventricular mass index (as determined by M‐mode echocardiography); and RWT1, relative wall thickness.

**Figure 2:**
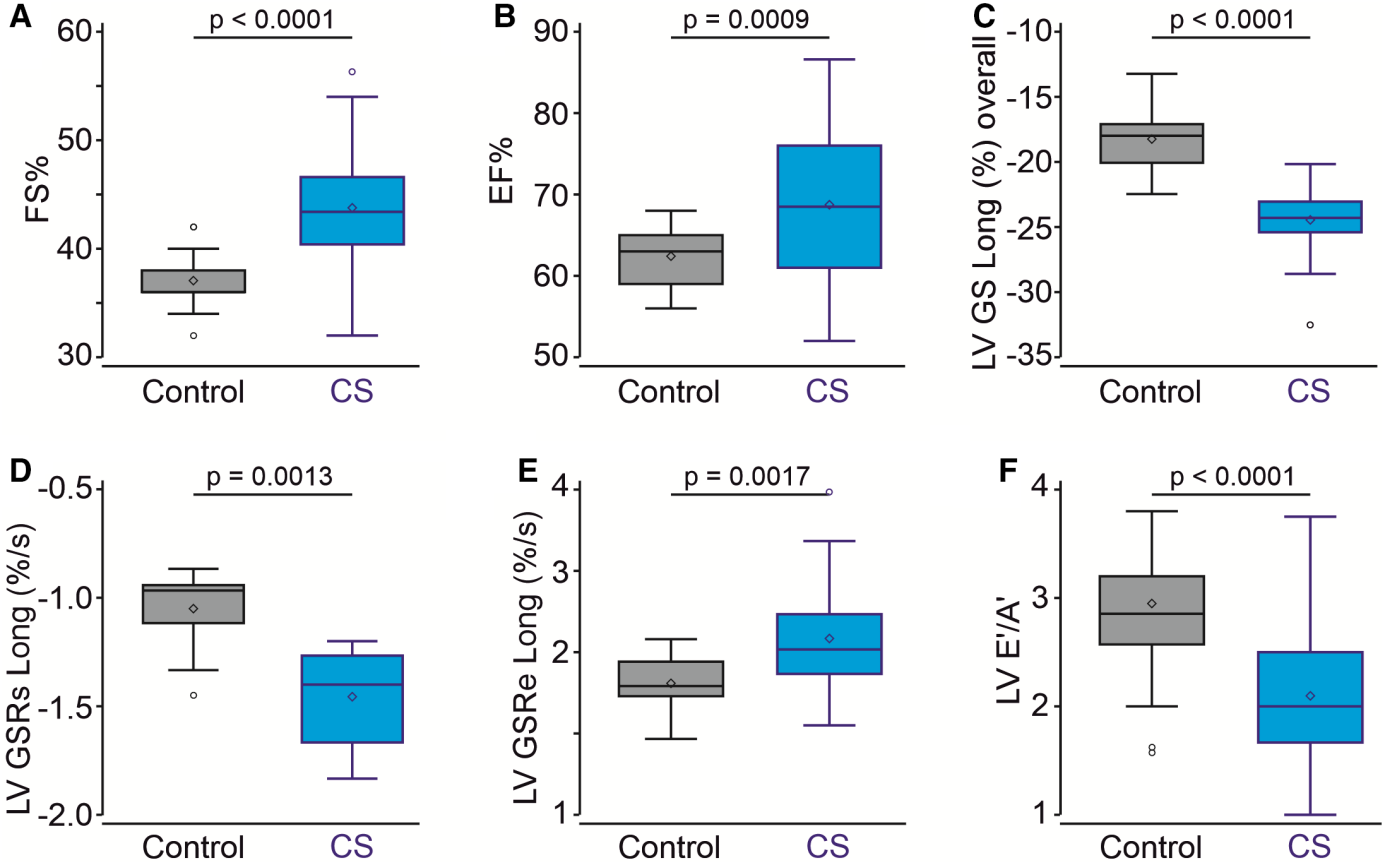
Enhanced left ventricular function in CS. Left ventricular FS (%) (**A**) and EF (%) (**B**) are increased in subjects with CS compared with the control group. LV GS Long overall (**C**), LV GSRs Long (**D**), and LV GSRe Long (**E**) are increased in subjects with CS. **F**, E'/A' is decreased in subjects with CS. *P* values from Wilcoxon rank sum tests are shown. *P*<0.05 indicates a statistically significant difference between the control and CS cohorts. A' indicates late diastolic velocity of mitral annulus; CS, Cantu syndrome; E', early diastolic velocity of mitral annulus; E'/A', ratio of E' over A'; EF, ejection fraction; FS, fractional shortening; LV, left ventricle; LV GS Long overall, LV global longitudinal systolic strain; LV GSRe Long, LV diastolic global strain rate; and LV GSRs Long, LV systolic global strain rate.

Subjects with CS demonstrated enhanced LV fractional shortening (Figure 2A) and ejection fraction (Figure 2B). In addition, LV contractility was significantly increased, with elevated LV global longitudinal systolic strain (Figure 2C) and systolic strain rate (Figure 2D). LV global longitudinal diastolic strain rate was marginally increased, and myocardial velocity indices were proportionally altered (Figure 2E and 2F, Table 2).

Because of the limited availability of RV‐focused 4‐chamber images, RV dimensions were only measurable in 15 of 31 subjects with CS and 10 of 17 control subjects. There was no significant difference (*P*=0.19) between the subjects with CS and the controls in median and quartile RV basal (2.8 cm; 2.5–3.9 cm versus 3.0 cm, 2.8–3.9 cm) and mid (1.8 cm; 1.5–2.5 cm versus 2.0 cm, 1.8–2.9 cm) axes, and longitudinal axis (4.8 cm; 4.5–7.2 cm versus 5.2 cm, 5.0–7.5 cm). The median RV annular systolic velocity S′ in subjects with CS were similar to those in the controls (12 cm/s versus 11.5 cm/s).

### Hemodynamics: High CO With Low Systemic Vascular Resistance

**Figure 3:**
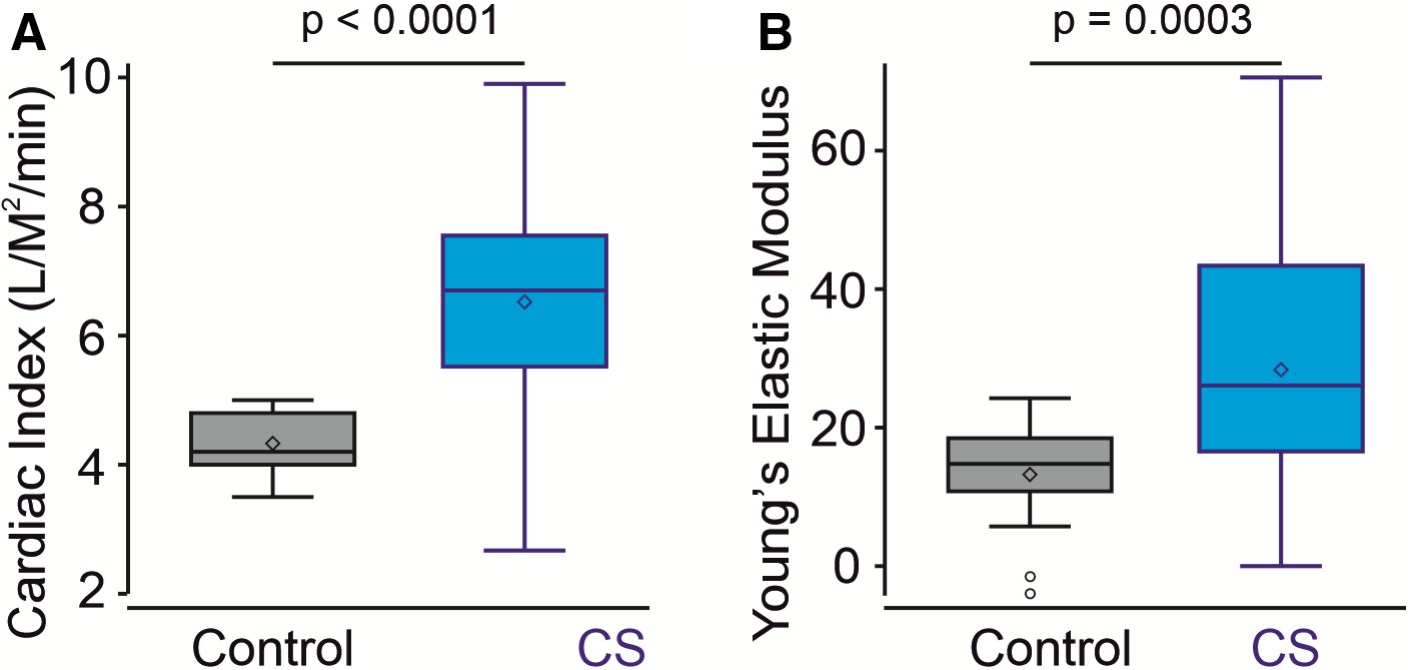
Hemodynamic metrics in subjects with CS. Cardiac index (cardiac output normalized to body surface area) (**A**) and vascular compliance (as measured by Young's elastic modulus) (**B**) are increased in subjects with CS. *P* values from Wilcoxon rank sum tests are shown. *P*<0.05 indicates a statistically significant difference between the control and CS cohorts. CS indicates Cantu syndrome.

Subjects with CS consistently exhibited significantly higher cardiac indices and decreased peripheral systemic vascular resistance than controls, and this was associated with significantly increased vascular compliance and diminished vascular tone (Figure 3, Table 2). Five subjects who were additionally assessed by cardiac catheterization (CS001, CS002, CS004, CS005, and CS020) displayed further evidence of high CO (cardiac index of 5.2–8.75 L/min^−1^ per m^−2^, respectively), with normal Left ventricular end diastolic pressure (7–9 mm Hg) and capillary wedge pressure (9–11 mm Hg). Systemic BP in individuals with CS assessed during the clinic (age range, 3–48 years) were low (Table 2) despite elevated CO, consistent with markedly decreased SVR and increased vascular compliance. Despite this, no subjects demonstrated HR or BP changes on standing.

### Pulmonary Hemodynamics

PAAT and PAAT/RV ejection time were in the normal range (>115 ms and 0.35 in adults, n=12; and >95 ms and 0.42 in children, n=19, respectively), suggestive of normal pulmonary systolic pressure and vascular resistance at initial presentation and during follow‐up.

### Characteristic Abnormalities of Valves and Great Vessels: Aortic Ectasia and Aortic Regurgitation

Almost all CS individuals (29/31, 94%) had aortic ectasia with dilation of the aortic root (*Z* score, 3.22–3.98) and ascending aorta (*Z* score, 2.16–3.57) at the first visit. In the remaining 2 subjects, aortic root dilation developed within 2 years follow‐up. No subjects with CS reached aortic dimension ≥5 cm, a cutoff point used for surgical aortic graft replacement in subjects with connective tissue disorders such as Marfan syndrome. Trivial or mild, and hemodynamically nonsignificant, aortic regurgitation was observed in some subjects, but there was no evidence of progression during the course of follow‐up. This was not associated with abnormal valve anatomy but was associated with aortic root dilation >3 *Z* score.

### Features of Fetal Circulation

Fifteen of 31 subjects with CS (48%) of all ages had features of fetal circulation that persisted, including small aorto‐pulmonary bronchial arteries in the thorax that were evident by color‐flow Doppler mapping. The most clinically pertinent feature of fetal circulation was the PDA present in 9 of 31 (29%) subjects with CS persistent beyond 2 months of age.

### Pericardial Effusion Complicating Clinical Course

Six of 31 subjects with CS (19%) had a pericardial effusion. A mother (CS004) and her daughter (CS002) developed pericardial effusion before the age 19 years and 9 years, whereas the 4 other subjects (CS011, CS016, CS021, and CS037) developed pericardial effusion in adulthood.

#### Electrocardiographic Monitoring

ECGs (n=31) revealed first‐degree atrioventricular block in 6 subjects, fascicular block in 2 subjects, and T‐wave axis abnormalities in inferior leads in 8 subjects, but there was no evidence of QT shortening or corrected QT prolongation, nor of atrial or ventricular arrhythmia. None of the subjects with CS met the voltage criteria for RV hypertrophy or LVH.

### Natural History

Four of 31 subjects with CS have been followed longitudinally from early childhood (CS001, CS002, CS003, and CS005) and 1 from early adulthood (CS004) through routine clinical assessments as a part of standard of care for a median of 9 years before systematic follow‐up in the CS research clinic over the ensuing 7 years. The other 26 subjects with CS were studied longitudinally in the CS research clinic for a median of 3 years (range, 2–7 years).

#### Cardiac Structure and Function

**Figure 4:**
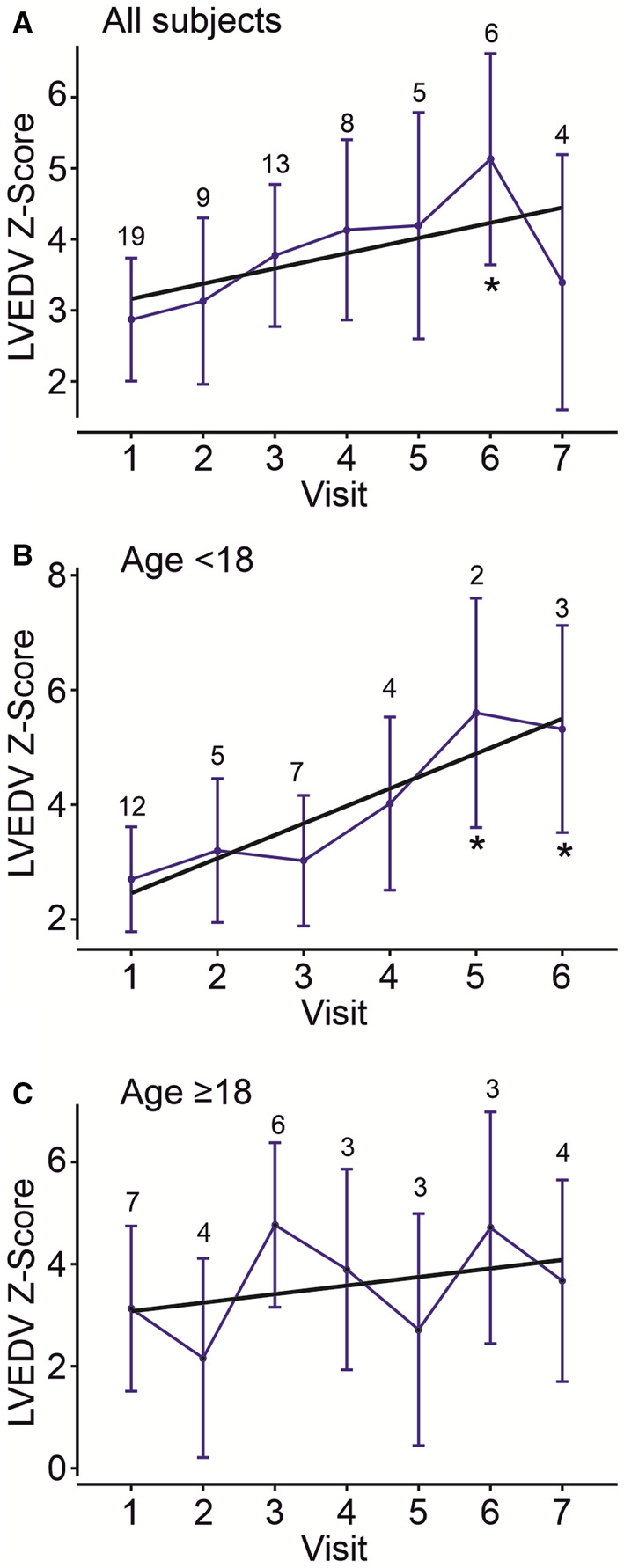
Longitudinal assessment of left ventricle dimension. LVEDV *Z* score was determined in the cohort of subjects with CS over up to 7 years. A trend toward increasing LVEDV *Z* scores was observed for all subjects with CS (**A**), and this was more pronounced for younger subjects (first visit at age <18 years [**B**]) than older subjects (first visit at age >18 years [**C**]). Data are shown as median *Z* score value for the CS cohort per visit (blue circles with 95% CIs; linear regression fits in black; slope=0.26, *P*=0.0609 [**A**]; slope=0.39, *P*=0.0208 [**B**]; slope=0.15, *P*=0.3652 [**C**]). Number of subjects at each visit is shown above data points. Data were fit with a mixed random‐effects repeated measures model to test whether changes across time were significant. *P<0.05 for any visit compared with visit 1. CS indicates Cantu syndrome; LV, left ventricular; and LVEDV, left ventricular end diastolic volume.

**Figure 5:**
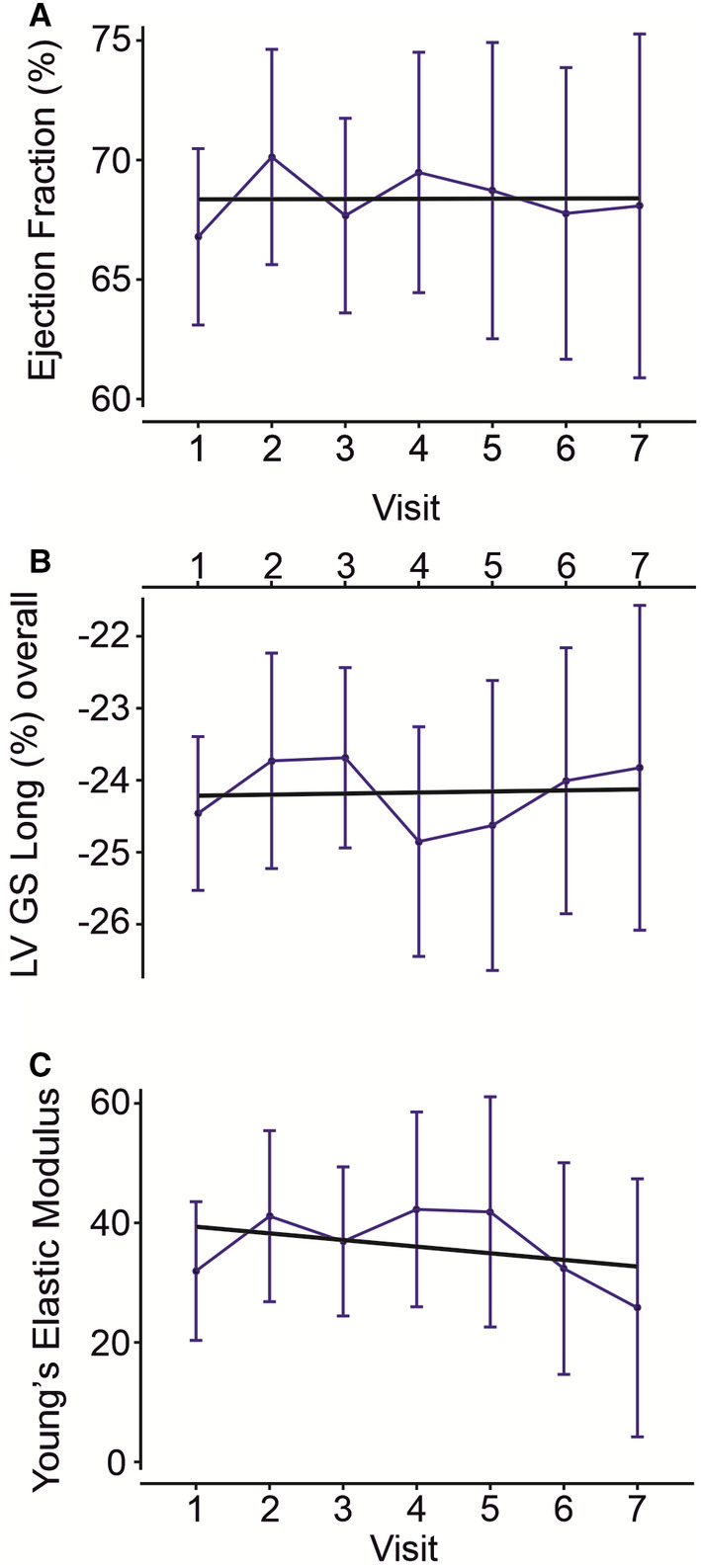
Longitudinal assessment of ventricular function and vascular elasticity. **A** and **B**, Both left ventricular ejection fraction (**A**) and LV GS Long overall (**B**) remained stable over repeated annual visits for subjects with CS. Number of subjects included in each repeat visit is indicated. Data are shown as median *Z* score value for the CS cohort per visit (blue circles with 95% CIs; linear regression fit shown in black; slope=0.85, *P*=0.06 [**A**]; slope=0.14, *P*=0.34 [**B**]). **C**, Young's elastic modulus did not significantly change over repeated annual visits for subjects with CS. Data are shown as median *Z* score value for the CS cohort per visit (blue circles with 95% CIs; linear regression fit shown in black; slope=−0.25, *P*=0.8755). Data were fit with a mixed random‐effects repeated measures model that showed there were no statistically significant differences in values over time. CS indicates Cantu syndrome; LV, left ventricular; and LV GS, left ventricular systolic global strain rate.

LV size increased gradually in all subjects with CS (Figure 4A) but more significantly in children and teenagers (Figure 4B and 4C). For the whole cohort, enhanced LV systolic function (ejection fraction; Figure 5A) and contractility (LV global longitudinal strain; Figure 5B) remained relatively stable over 7 years of follow‐up.

#### Hemodynamics

The abnormally high vascular compliance remained relatively unchanged over time (Figure 5C). Overall, systemic vascular resistance remained low, and cardiac index remained high for the whole cohort during follow‐up period. The majority of subjects with CS remained in New York Heart Association functional class I, although 4 of 31 (CS004, CS021, CS035, and CS022), representing 4 of the 5 women aged ≥40 years, exhibited evidence of symptomatic HF. CS004 developed fatigue, shortness of breath on climbing stairs, and orthopnea (New York Heart Association functional class III) at the age of 42 years and before the onset of atrial fibrillation, which worsened the HF severity (brain natriuretic peptide, 200 pg/mL). Treatment with an angiotensin‐converting enzyme inhibitors, prescribed by her treating cardiologist at the time for HF management, exacerbated symptoms, and was later discontinued. The 3 other subjects (CS021, CS035, and CS022) developed HF (all New York Heart Association functional class II) at age 46, 58, and 69 years, respectively, with increasing symptoms of fatigue, orthopnea, and exercise intolerance, and were treated with mild diuretics. Although all other subjects with CS were in New York Heart Association functional class I, they generally complained of fatigue and intolerance to moderate‐ or high‐intensity exercise. Three additional subjects (CS005, CS006, and CS0020, aged 23, 17, and 20 years, respectively) had orthopnea, but became symptom free after they were taken off an angiotensin‐converting enzyme inhibitor that was prescribed by their treating physicians because of cardiomegaly.

#### Pericardial Effusion

One mother (CS004) and her daughter (CS002) developed hemodynamically significant recurrent pericardial effusion at 19 years and 9 years of age, respectively, that required repeated pericardiocentesis and ultimately pericardial stripping. The pericardial fluid had an elevated protein concentration (4.7 g/dL and 4.4 g/dL), normal glucose (90 mg/dL and 95 mg/dL), and predominant lymphocyte population on cell count (≈70%) in the presence of negative inflammatory markers (erythrocyte sedimentation rate <10 mm/h, C‐reactive peptide <0.1 mg/L, negative serum antiextractable nuclear antigen antibody, and normal C3 and C4 complements), suggesting that the effusion might be lymphatic pericardial fluid. Four other subjects (CS011, CS016, CS021, and CS037) had stable and asymptomatic mild‐to‐moderate pericardial effusion during follow‐up.

#### Features of Fetal Circulation

Subjects with CS with aorto‐pulmonary collaterals were asymptomatic, except for 1 subject (CS020, aged 20 years) with a large aorto‐pulmonary bronchial artery who developed hemoptysis and required embolization of the vessel by cardiac catheterization. Compared with preintervention, the postintervention follow‐up *Z* score of left ventricular end diastolic volume indexed (2.8 versus 2.7), and left ventricular end diastolic dimension (2.5 versus 2.4) as well as LVMI (88 versus 86 g/h^2.7^) and cardiac index (6.5 versus 6.9 L/min per m^2^) were relatively unchanged. In 9 subjects with persistent PDA beyond 2 months of age, 6 had a hemodynamically significant shunt requiring surgical or device closure before 1 year of age, and before their enrollment in the study. They all had enlarged left ventricles (LV end‐diastolic volume *Z* score, 2.9–3.3) and increased cardiac index (6.7–7.5 L/min per m^2^) even a median of 3 years (range, 2–7 years) after the closure of the PDA. Taken together, in these subjects with CS, increased CO, and enlarged left ventricle were predominantly associated with low SVR and high output.

## DISCUSSION

**Figure 6:**
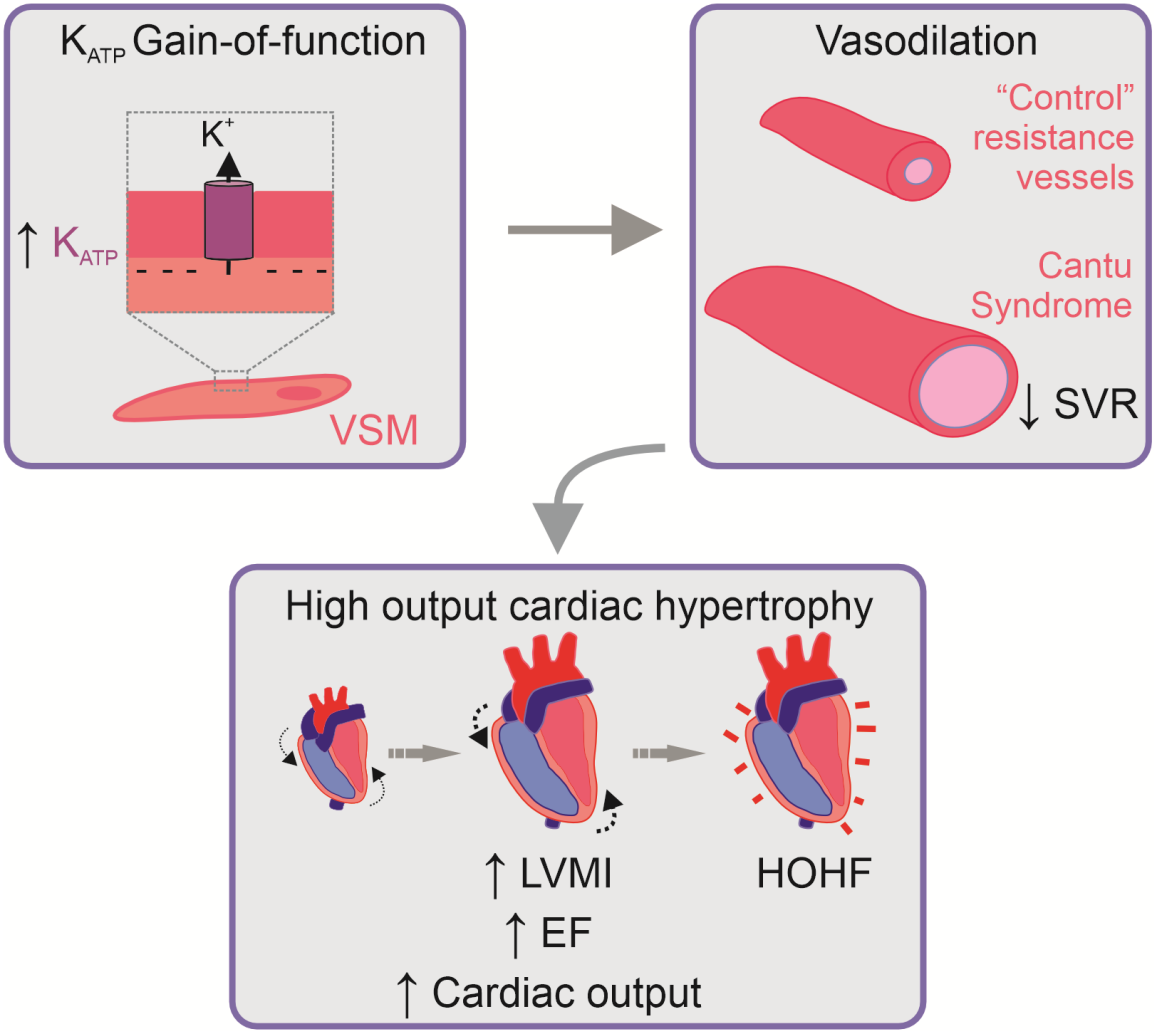
Mechanism of cardiovascular pathophysiology in CS. (Top left) K_ATP_ channel composed of Kir6.1 (*KCNJ8*) and SUR2 (*ABCC9*) is expressed in the plasma membrane of smooth muscle cells, including VSM. CS‐associated *ABCC9* or *KCNJ8* variants cause gain‐of‐function of K_ATP_ channels in VSM, driving hyperpolarization of the membrane potential. (Top right) VSM membrane potential hyperpolarization is predicted to result in chronic vasodilation in CS, as reflected by marked decreases in SVR. (Bottom) Cardiac remodeling to high‐output hypertrophic state arises secondary to chronically lowered SVR, resulting in elevated cardiac mass, contractility, and output in subjects with CS. This progresses to HOHF in older subjects. CS indicates Cantu syndrome; EF, ejection fraction; HOHF, high‐output heart failure; K_ATP_, ATP‐sensitive potassium; LVMI, left ventricular mass index; SVR, systemic vascular resistance; and VSM, vascular smooth muscle.

We describe a specific cardiovascular phenotype resulting from gain‐of‐function variants in *ABCC9* (SUR2) that presents with dilated left ventricle with increased LV mass, but increased contractility and high CO, associated with genetically determined primary low systemic vascular resistance. Although SUR2 proteins are regulatory subunits of both cardiac and vascular smooth muscle K_ATP_ channels,^25^ we see no evidence of cardiac arrhythmias, and essentially none were observed during 24 hours of ambulatory ECG monitoring in a previous study.^10^ That study revealed generally high HR for age that failed to lower appropriately during sleeping hours, suggesting a relatively elevated sympathetic activity but diminished vagal activity. Mechanistic studies in mouse models of CS, in which CS variants in *ABCC9* and *KCNJ8* were engineered into the equivalent mouse loci using CRISPR/Cas9, indicate that cardiac enlargement and enhanced CO are responses to decreased vascular resistance arising from the vascular smooth muscle K_ATP_ channel gain‐of‐function, because cardiac enlargement can be reversed when vascular K_ATP_ channels specifically are inhibited or genetically knocked down^26^, ^27^ (Figure 6). Thus, the cardiac hypertrophy and high‐output state in CS may be a consideration for causes of HF but are distinct in phenotype, hemodynamic, and natural history from cardiomyopathies of known causes, including HF with preserved ejection fraction.

The CS phenotype is distinct from dilated cardiomyopathy, in which the ventricles are enlarged but thin walled, and systolic function is decreased, and from hypertrophic cardiomyopathy, in which ventricular walls are thickened but chamber cavity is either normal or smaller than normal. Unlike CS, both dilated cardiomyopathy and hypertrophic cardiomyopathy may have abnormal diastolic indexes of LV filling and diastolic function.^28^ Systematic evaluation of CS cardiovascular features has previously been lacking, and the combination of dysmorphic features and cardiomegaly has previously led to misdiagnoses of subjects with CS as having a metabolic storage disorder or other genetic syndrome, or dilated cardiomyopathy or hypertrophic cardiomyopathy, leading to inappropriate and potentially harmful therapeutic choices. CS is also distinct from other high‐output conditions in which low SVR is secondary to the underlying cause. For example, high‐output cardiac failure associated with the hyperdynamic phase of sepsis is mainly driven by inflammatory cytokines, which cause systemic vasodilation in the setting of otherwise normal myocardial function. Severe thiamine deficiency (Beriberi), in which buildup of pyruvate and lactate in the blood leads to systemic vasodilation, also results in a high CO state and cardiomyopathy. Low SVR and high‐output failure may also be secondary to many other conditions, including arteriovenous malformation, liver disease, myeloproliferative disorders, acromegaly, and Paget disease of bone, but associated clinical features distinguish them from CS.^29^ High‐output states are commonly detrimental to right heart structures and function, but in the few subjects with CS, in which RV function was assessed, this was not apparent. High‐output cardiac hypertrophy in CS may compete in differential diagnosis for HF with preserved ejection fraction. However, HF with preserved ejection fraction is essentially a diastolic HF condition, whereas diastolic function is essentially normal in CS.

Athlete's heart syndrome, associated with eccentric enlargement as a consequence of repetitive cardiac loading,^30^ is a superficially similar cardiac phenotype. Notably, athlete's heart syndrome is generally considered benign, without physical symptoms, other than persistently low resting heart rate.^31^ CS is associated with additional cardiovascular issues that are not present in athlete's heart syndrome, but which could also feature in other high‐output hypertrophic states. Features of fetal circulation, such as persistent PDA and dilated and tortuous collateral arteries in general, are common findings in subjects with CS,^32^, ^33^ and are consistent with the proposed basis of the cardiac phenotype as a response to decreased SVR.

CS thus represents a sentinel example of a secondary cardiac condition that could, in principle, arise from a primary abnormal vasodilation of any cause. The long‐term prognosis of this high‐output cardiac hypertrophy is not clear. Interestingly, in the only large‐scale analysis of subjects with high‐output HF of various causes, both high output and mortality were strongly correlated with the severity of decreased SVR, and outcomes were poorest among subjects with the lowest SVR.^29^ These findings do suggest that low SVR and the consequent high‐output hypertrophic cardiac response may not be benign. We would predict that the condition should be associated with a decreased reserve in terms of ability to respond to additional demands, and the 7 years of our systematic study reveal a trend toward worsening clinical HF status. Additionally, 4 of the 5 subjects who were aged >40 years, all women, showed signs of high‐output HF. High‐output HF^29^, ^34^ exhibits similar features to the cardiovascular phenotype in CS (including decreased exercise tolerance, low SVR, and elevated CO). CS may therefore represent a genetically defined basis for high‐output HF.

A full understanding of the long‐term cardiovascular manifestations of CS, facilitated by recognition of the underlying molecular cause, may have clearer significance for potential therapeutic approaches. Because of the current lack of awareness of CS complications, many subjects with CS have been misdiagnosed with hypertrophic cardiomyopathy or dilated cardiomyopathy, and as a result have been administered conventional therapies including angiotensin‐converting enzyme inhibitors or other vasodilatory agents, diuretics, β‐blockers, or sildenafil.^9^ Treatment with these agents are based on HF paradigms that do not take into account low SVR states and will exacerbate the underlying primary decrease in SVR and hence worsen symptoms. Ultimately, an appropriate therapy for CS, and potentially for other causes of high‐output cardiac hypertrophy caused by decreased SVR, will address the underlying primary vasorelaxation. In the specific case of CS, this might ideally be a blocker of the overactive K_ATP_ channels themselves. Animal studies have shown that the K_ATP_ channel inhibitor glibenclamide reverses the primary decrease in SVR, and hence the secondary cardiac enlargement,^35^ raising the possibility that this approach may be effective, by treating the underlying defect directly.

### Limitations of the Study

CS is rare and underdiagnosed, limiting the available sample size for this study. Presentation of the characteristic cardiac phenotype and natural history may help improve diagnosis of CS, and identification of the relevant cardiac features. In the majority of cases, hemodynamics were primarily determined by noninvasive tests rather than by cardiac catheterization. However, limited validation in a few subjects with CS confirmed the accuracy of noninvasive‐derived hemodynamic data.

## Sources of Funding

This work was supported in part by National Institutes of Health grants R21 HD103347 (Eunice kennedy shriver national institute of child health & human development to D.K.G. and C.G.N.) K99 HL150277and R35 HL140024 (National heart, lung, and blood institute to C.G.N. and C.M.), and the Children's Discovery Institute (CH‐MD‐II‐2015‐488 to C.G.N) and the Center for the Investigation of Membrane Excitability Diseases Pilot and Feasibility program (CIMED‐14‐03 to D.K.G.). C. McClenaghan is supported by a National Institutes of Health grant K99 HL150277.

## Disclosures

None.
